# A Thermal, Mechanical, and Materials Framework for a Shape Memory Alloy Heat Engine for Thermal Management

**DOI:** 10.3390/nano13152159

**Published:** 2023-07-25

**Authors:** Maria Chikhareva, Raj Vaidyanathan

**Affiliations:** Department of Materials Science and Engineering, University of Central Florida, Orlando, FL 32816, USA; maria.chikhareva@knights.ucf.edu

**Keywords:** shape memory alloy, NiTi, Nitinol, heat engine, thermal management, Stirling

## Abstract

Shape memory alloy (SMA) heat engines possess an inherent property of sensing a change in temperature, performing work, and rejecting heat through the shape memory effect resulting from a temperature-induced phase transformation. This work presents a framework for the design and implementation of an SMA-based Stirling heat engine for maximum torque or speed incorporating and combining mechanical, thermal, and material aspects. There is a growing need for such engines for reliable thermal management and energy recovery in both ground and space applications. Mechanical aspects were addressed from force balances in the SMA element and focused on the resulting stress distribution. Thermal aspects considered heat transfer between the SMA element and both the heat source and the heat sink. Materials aspects considered the chemical, elastic, and frictional contributions to the enthalpy of the transformation. The roles of nano- and microstructure through composition, precipitates, variant interfaces, training, cycling, texture, defects, nucleation sites (bulk vs. surface), and multi-step transformations (e.g., a trigonal R-phase transformation) in NiTi based-alloys are also emphasized. The aforementioned aspects were combined to present a figure of merit to aid in the design and implementation of a Nitinol Stirling heat engine operating to maximize torque or maximize speed.

## 1. Introduction

Near equi-atomic NiTi is a commonly used shape memory alloy (SMA) where the transformation occurs near room temperature between a low symmetry, B19’ monoclinic, so-called martensite phase and a high symmetry, B2 cubic, so-called austenite phase upon heating. This transformation can occur under high stresses (nominally around 600 MPa uniaxially) while concomitantly recovering high strains (nominally up to 8% uniaxially) [1,2]. Cooling results in the austenite phase returning to martensite which deforms at lower stresses than the parent austenite phase. The forward transformation from austenite to martensite is exothermic while the reverse transformation from martensite to austenite is endothermic. These two important features—one, the difference in the stiffness between the austenite and martensite phases, and two, the ability to absorb and dissipate heat (i.e., endothermic and exothermic transformations)—make them viable for use as materials that are heat engines. The aforementioned force-generating phase transformation is in response to changes in temperature, and thus, the SMA inherently acts as both a sensor and an actuator. Furthermore, SMAs have high force output-to-weight ratio in comparison with traditional phase change materials [1,2]. Thus, SMA heat engines are appealing in applications where requirements call for compact designs (e.g., without external sensors and additional power sources), in cases where energy can be scavenged from the ambient environment.

Ternary alloy elemental additions to Ni and Ti such as Fe, Co, and Nb can shift the transformation to cryogenic temperatures [1] while elements such Hf and Pt can increase the transformation to above room temperatures [3]. Increasing their temperature range of operation makes SMA thermal management devices of interest in zero boil-off systems for cryogenic liquefaction and densification on the moon and Mars in addition to terrestrial heat harvesting and recovery systems. There is such a need for reliable thermal management and energy recovery in both terrestrial and space applications [4,5].

While SMA heat engines have previously been proposed and demonstrated [6], there has been a lack of a systematic approach in the comprehensive analysis of SMA heat engines incorporating materials, mechanical, and heat transfer aspects. Their ability to function in environments where they scavenge “free” heat that would nominally be wasted has not been utilized. The framework presented here builds on a previous body of work that has considered SMA thermal switches operating in a make or break contact mode [7,8,9,10,11].

The extension here is to consider aspects of an engine in continuous operation where the SMA is the heat engine as opposed to being coupled with an external device to transfer heat (e.g., heat pipe in [7]). The intent of this work is twofold. First, it is to establish a framework for designing and implementing an SMA heat engine uniquely combining materials, mechanical, and heat transfer aspects. Second, it is to examine conditions for optimized performance based on the targeted application. Previous work (e.g., [6,12]) has concentrated on estimating the maximum work output and thermal efficiency of the SMA heat engine and did not take into consideration additional benefits, e.g., high speed of engine operation or torque. The framework proposed distinguishes between these modes of performance.

The nanomaterials aspect draws on current understanding of the role of nano- and microstructure in the thermoelastic martensitic transformation at the nanoscale and how the nano- and microstructure can be optimized for SMA heat engine performance. Emphasis is placed on engineering future SMA heat engine materials starting at the nanoscale by alloy selection and thermomechanical processing, making recommendations vis-á-vis the size and distribution of precipitates, variant interfaces through texture, cycling and training, defects, nucleation sites (bulk vs. surface), and multi-step transformations (e.g., a trigonal R-phase transformation).

## 2. SMA Stirling Engine

### 2.1. Efficiency

**Figure 1:**
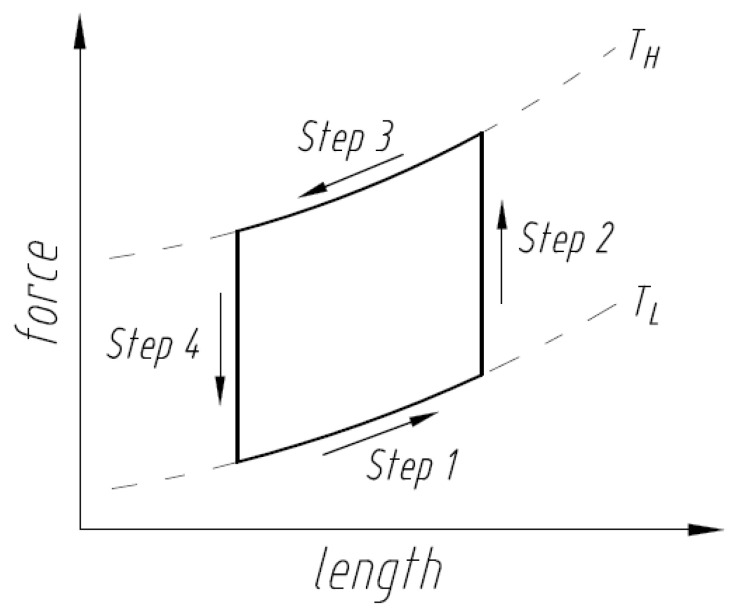
Work cycle diagram of an SMA Stirling engine.

One type of heat engine is a Stirling engine which employs four steps in its reversible cycle when a gas is the working medium: isothermal expansion, isothermal contraction, and two steps with constant volume during which the pressure of the gas increases or decreases as a result of an external change in temperature. The Stirling engine uniquely exhibits an efficient reversible heat engine cycle that can be practically implemented while incorporating two constant volume (or constant length) steps with changing pressure (or force) steps, compared to other practical engines (e.g., Ericsson) [13]. When an SMA element is used as a force generating element in a Stirling engine, the ability of the engine to perform work is based on the intrinsic property of the SMA element to recover stress under restrained conditions. As shown in Figure 1, in Step 1 a load is applied to the SMA element in the martensite state (at low temperature $T_{L}$) which causes an increase in length due to movement of twin-related martensite boundaries (martensite detwinning). An increase in temperature in Step 2 to high temperature $T_{H}$ causes the martensite to revert to the austenite phase. The increase in stiffness of the austenite compared to the detwinned martensite results in a decrease in length in Step 3. Step 4 reverts austenite to martensite with a drop in temperature from $T_{H}$ to $T_{L}$, and the engine continues to cycle.

The maximum Carnot theoretical efficiency of a Stirling engine $\eta_{Carnot}^{max}$ as a ratio of the difference between the high and low engine operating temperatures to the high temperature, independent of the working medium, was estimated to be 35.6% [13]:

(1) $$\eta_{Carnot}^{max}=\frac{T_{H}-T_{L}}{T_{H}}=35.6\%$$

However, the thermal efficiency of a heat engine with an SMA element $\eta_{SMA}$ as high as 12% has been reported from the ratio of the work done by the engine per cycle $W_{out}$ to the heat taken in per cycle $Q_{in}$ [12]:

(2) $$\eta_{SMA}=\frac{W_{out}}{Q_{in}}$$

Even though the thermal efficiency of an SMA Stirling engine is much lower than its maximum Carnot efficiency, the ability of the SMA to perform work that can be activated by changes in ambient temperature or even by waste or parasitic heat, utilizing free and accessible energy, is advantageous. Therefore, comparisons of absolute values of the Carnot efficiency or thermal efficiency may not be relevant for evaluating SMA heat engines.

### 2.2. Configuration

**Figure 2:**
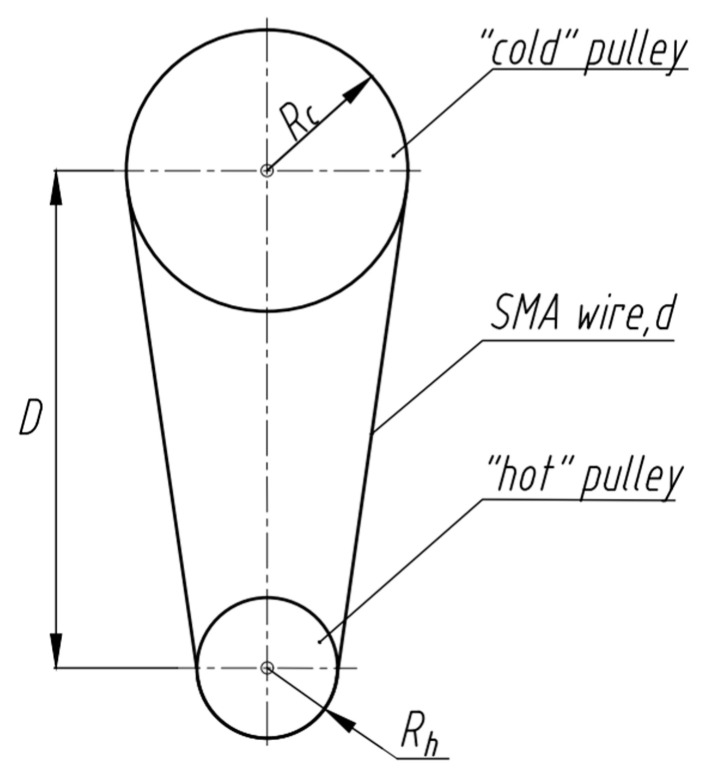
Commercially available unsynchronized pulley type SMA Stirling engine demonstration considered in this work.

The framework adopted here considers a Stirling cycle heat engine of an unsynchronized pulley type as shown in Figure 2. Such an engine offers the opportunity to examine the fundamental underlying materials, mechanical, and thermal aspects while being easily amendable to other types. Figure 2 shows relevant variables based on a commercially available demonstration (Sci-Supply, Fairfield, OH, USA). The engine consists of two pulleys of different radii (radius of the “hot” pulley with the wire there in equilibrium with the heat source $R_{h}$ and radius of the “cold” pulley with the wire there in equilibrium with the heat sink $R_{c}$) and an SMA wire diameter d. The distance between the pulleys is D. The nominal dimensions for $R_{h}$, $R_{c}$, D, and d were 15, 43, 185, and 0.45 mm, respectively. The SMA wire (nominal composition 48.5 at% Ni 51.5 at% Ti) had transformation temperatures of austenite start $A_{s}$, austenite finish $A_{f}$, martensite start $M_{s}$, and martensite finish $M_{f}$ equal to 69, 84, 58, 44 °C, respectively, and operated between hot water at 90 °C and the ambient laboratory temperature set at 22 °C. The wire was shape set at 525 °C for 25 min to a straight shape and then fixed in a loop. While the purpose of this work was to present a theoretical framework of an SMA heat engine, the working demonstration was used to qualitatively identify and validate important issues as presented later in this work.

## 3. Analyses

### 3.1. Mechanical and Thermal Analyses

The stress and strain distribution in the SMA element resulting from changes in temperature are unique for a given engine design and ultimately determine the engine output. For the case of the unsynchronized pulley type engine considered here, load transfer between the independently rotating pulleys is enabled by friction between the pulleys and the SMA wire that loops around them. Forces are transmitted from the driving pulley (the pulley of smaller diameter in contact with the heat source in Figure 2) to the wire and then from the wire to the driven pulley (the pulley of larger diameter in contact with the heat sink in Figure 2). The engine performs work as the mechanically bent SMA wire straightens upon heating. In the static state (no rotation, constant temperature), a preload force and bending force resulting from the pulleys exist in the wire.

The macroscopic strains in the SMA wire due to bending are a function of pulley radii and wire diameter. The bending strain in the wire around the hot pulley $\varepsilon_{h}$ is higher than the strain around the cold pulley $\varepsilon_{c}$, and these strains are respectively given by

(3) $$\varepsilon_{h}=\frac{d}{2R_{h}+d}$$

(4) $$\varepsilon_{c}=\frac{d}{2R_{c}+d}$$

The strains lead to tensile stresses in the outer section of the wire and compressive stresses in the inner section.

From Figure 1, an SMA Stirling engine operates between temperatures $T_{L}$ and $T_{H}$ which include the temperatures at which the forward transformation to martensite starts and the reverse transformation to austenite finishes. Through the reverse transformation in Step 2, torque $T_{h}$ is generated at the hot pulley, which results in a circumferential force $F_{c}$ from

(5) $$F_{c}=\frac{T_{h}}{R_{h}}$$

**Figure 3:**
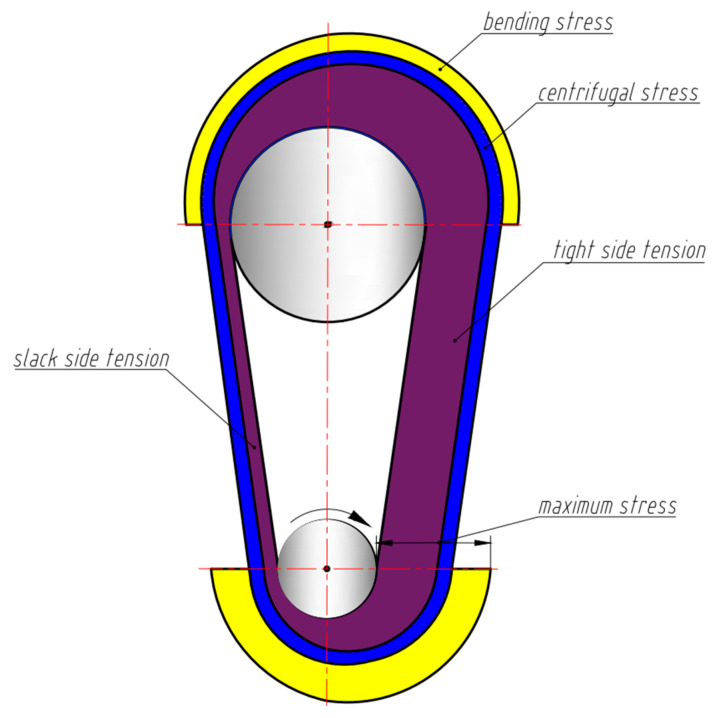
The total stress distribution profile in the SMA heat engine while running clockwise.

This circumferential force results in different forces in the two wire sections on either side of the pulley and forms a tight (leading) and a slack (trailing) side with respect to the direction of rotation in Figure 3, with force $F_{S}$ on the slack side and force $F_{T}$ on the tight side, respectively. This analysis was also qualitatively verified using the demonstration engine. The final forces then depend on the preload $F_{init}$ (Step 1 in Figure 1) and the circumferential force $F_{c}$:

(6) $$F_{T}=F_{init}+\frac{F_{c}}{2}$$

(7) $$F_{S}=F_{init}-\frac{F_{c}}{2}$$

If the engine operates at low speed, the forces at constant torque can be considered to be independent of the angular speed of the hot pulley. However, at high speeds, centrifugal forces act on the wire section which runs around the pulleys. The resultant centrifugal force pushes the wire outwards and lifts it off the pulley resulting in additional tension. It causes a reduction in the frictional force between the wire and pulleys and, ultimately, can cause slippage [14].

The SMA performs work by recovering the mechanically induced deformation to its shape-set shape. Specifically for the unsynchronized pulley engine configuration, the mechanically bent SMA wire straightens upon heating and, therefore, generates torque to run the engine. When the wire exchanges heat with the source (the temperature of heat source $T_{H}>A_{f}$) in Step 2 (Figure 1), the transformation from martensite to austenite is initiated which causes the SMA in Step 3 to recover strain under a constraint and to return to the initial shape. In Step 4, as the wire exits the heat source, it cools due to the heat sink (the temperature of heat sink $T_{L}<M_{f}$) to a temperature at which the transformation to martensite is complete or to a temperature below it. As the SMA wire returns to martensite, the transformation stress relaxes, but the stress in the wire from looping around the pulley from Step 1 remains. This cycle repeats to result in a running engine. The maximum stress $\sigma_{max}$ can be determined from Equation (8) as a sum of the stresses associated with the force on the tight side $\sigma_{T}$, the bending recovery stresses at the hot pulley $\sigma_{BH}$, and the stress associated with the centrifugal force $\sigma_{CF}$ and is shown in Figure 3.

(8) $${\sigma_{max}=\sigma_{T}+\sigma_{BH}+\sigma }_{CF}$$

The engine output torque results from the difference between the bending recovery stress at the hot pulley and the biasing work. In the heat engine considered here, the biasing work to bend the wire over the cold and hot pulleys reduces the recovery stress of the SMA wire at the hot pulley.

**Figure 4:**
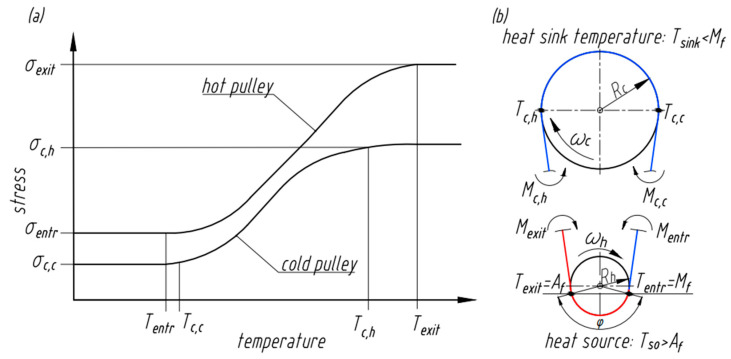
(**a**) Representative stress in the SMA wire with temperature, (**b**) four wire positions (prior to entering and immediately after exiting the hot and cold pulleys, respectively) where power is generated.

Figure 4a shows the stress vs. temperature response of a representative wire operating in the configuration under consideration. The bending moments, which are generated at four key positions (prior to entering and immediately after exiting the hot and cold pulleys, respectively) indicated in Figure 4b, result in output torque which can be expressed as follows [15]:

(9) $$\begin{array}{c} T_{out}=\frac{R_{c}}{R_{h}}\left(M_{exit}-M_{entr}\right)-\left(M_{c,h}-M_{c,c}\right)\\ =\frac{\pi d^{3}}{32}\left(\frac{R_{c}}{R_{h}}\left({\sigma_{exit}-\sigma }_{entr}\right)-\left({\sigma_{c,h}-\sigma }_{c,c}\right)\right) \end{array}$$

where $M_{entr},M_{exit},M_{c,h},$ $M_{c,c}$ and $\sigma_{\sigma_{entr}}$, $\sigma_{exit}$, $\sigma_{c,h},\sigma_{c,c}$ are bending moments and stresses acting over the wire cross section in the positions prior to entering, upon exiting the heat source, and at points of the first contact of the wire with the cold pulley (hot section of the wire) and the last contact (cold section), respectively.

**Figure 5:**
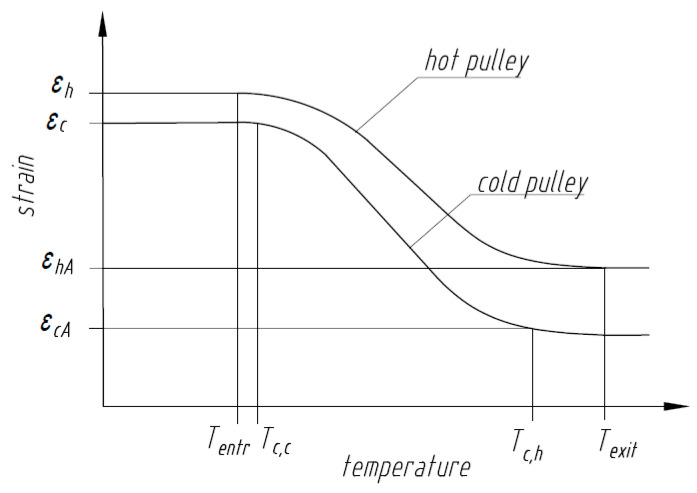
Representative strain in the SMA wire with temperature.

Figure 5 shows an alternative approach to estimating $T_{out}$ by considering strains in the SMA wire at different temperatures rather than the stress at each position where torque is generated in Figure 4b. Initially the SMA wire bent around the hot and cold pulleys experiences bending strains in accordance with Equation (1) and Equation (2), respectively. The SMA wire is under maximum deformation $\varepsilon_{h}$ at the hot pulley. When the wire is heated to $A_{f}$, the wire transforms from martensite to austenite recovering strain to $\varepsilon_{h_{A}}$. Upon exiting the heat source, the wire at temperature $T_{exit}$ cools down to $T_{c,h}$ and further to $T_{c,c}$ (Figure 4b). The wire transforms back to martensite and, at the cold pulley, undergoes deformation from a partially transformed martensitic state $\varepsilon_{c_{A}}$ to a completely transformed martensitic state $\varepsilon_{c}$.

At any temperature, the wire recovery strain at the hot pulley is larger than at the cold pulley, resulting in different curves for the strain vs. temperature response at the hot and cold pulleys.

In addition to the stress and strain distribution in the SMA wire, mechanical aspects also include design specifics such as SMA element shape and dimensions, number of SMA elements, external biasing work, etc. An appropriate way to examine the effect of the main mechanical variables collectively on the behavior of SMA elements and the performance of SMA Stirling engines is through the operational speed of the engine. For the general case, consider heat transfer between the SMA element and the heat source at temperature $T_{so}$. The initial temperature of the wire is $M_{f}$ or possibly below due to undercooling at the cold pulley. For convenience, $M_{f}$ is considered here since undercooling can be prevented by tailoring the dimensions of the pulley in order to reduce cooling. From the heat source, the wire is heated up to the transformation temperature $A_{f}$ or possibly higher. A similar argument can be used at the hot pulley or heat source to consider $A_{f}$ as the final temperature. Assuming the heat required by the SMA element is equal to the heat received from the source and applying Newton’s law of cooling, the time for this heat transfer, τ, can be expressed as follows:

(10) $$\tau =-\frac{\rho \cdot c_{p}\cdot d}{4h}\ln\left(\frac{T_{so}-A_{f}}{T_{so}-M_{f}}\right)$$

where $\rho ,\;c_{p}$, and h are density, specific heat, and heat transfer coefficient, respectively.

From the geometry of the unsynchronized pulley considered here and assuming a time for heat transfer from Equation (10), the angular distance travelled is φ (Figure 4b), and the angular speed of the hot pulley is

(11) $$\omega_{h_{max}}=-\frac{4\cdot h\cdot \varphi }{\rho \cdot c_{p}\cdot d\cdot ln\left(\frac{T_{so}-A_{f}}{T_{so}-M_{f}}\right)}$$

We note that from Equation (9), SMA elements of larger diameter produce higher torque but take longer time to transfer heat according to Equation (10) and result in slower angular speed in accordance with Equation (11) due to lower heat transfer.

In accordance with Equation (10), a low-temperature heat source requires longer time to heat up an SMA element and to transform the martensite to austenite. If the contact time between the SMA element and the heat source is not sufficient to accomplish the reversible transformation, the material will need more time to absorb heat required for the transformation. Therefore, in the case when the temperature of the heat source is only slightly above austenite finish temperature, $T_{so}>A_{f}$, the engine makes a self-correction by lowering the angular speed to increase the contact time with the heat source. In contrast, having a heat source at a temperature much higher than $A_{f}$ increases the heat transfer rate and decreases the transformation time. In accordance with Equation (11), heating of the SMA wire at $T_{so}\gg A_{f}$ is expected to drive the engine at higher speeds. Both these predictions were qualitatively validated using the demonstration engine.

In addition, the time spent for heat transfer and subsequent complete phase transformation at the hot and cold pulleys can be used to determine the overall geometry of the engine (i.e., pulley diameters, distance between the pulleys) in the framework.

### 3.2. Material Thermodynamics

Any sufficient temperature difference in thermal energy can drive an SMA Stirling engine. The required heat $Q_{in}$ to activate the transformation can be estimated based on SMA properties from [16]

(12) $$Q_{in}=Q_{s}+\Delta H_{net}{+W}_{SMA}$$

where $Q_{s}$, $\Delta H_{net}$, and $W_{SMA}$ are the sensible heat, stress-free transformation enthalpy change, and SMA work output, respectively. Change in any of these terms on the right side of the equation will affect the total amount of required heat.

The sensible heat is the amount of heat required to change the temperature of the SMA element from the martensite finish temperature to the austenite finish temperature. In addition to the temperature range, the amount of sensible heat depends on material density ρ, specific heat $c_{p}$, and stress in Step 1 and Step 3 at which the SMA element is operating (Figure 1). Due to the thermoelastic nature of the martensitic transformation in SMAs, external stress results in an increase in the transformation temperatures [1]. In accordance with the Clausius–Clapeyron equation, sensible heat for the SMA element operating under applied stresses $\sigma_{1}$ and $\sigma_{2}$ in austenite and martensite states, respectively, is given by

(13) $$Q_{s}=\rho \cdot c_{p}\cdot \left(A_{f}^{\sigma_{1}}-M_{f}^{\sigma_{2}}\right)=\rho \cdot c_{p}\left(A_{f}-M_{f}+\frac{\sigma_{1}-\sigma_{2}}{\frac{d\sigma }{dT}}\right)$$

where ${(A}_{f}-M_{f})$ is the difference between the stress-free austenite finish and martensite finish temperatures or the nominal hysteresis in the transformation while $A_{f}^{\sigma_{1}}$ and $M_{f}^{\sigma_{2}}$ are the temperatures of the austenite finish transformation under applied stress $\sigma_{1}$ and the temperature of martensite finish transformation under applied stress $\sigma_{2}$, respectively.

For a thermoelastic transformation, the stress-free enthalpy change can be given as [17]

(14) $$\Delta H_{net}=\Delta H_{ch}+\Delta H_{el}(T)+\Delta H_{i}(T)$$

where $\Delta H_{ch}$, $\Delta H_{el},$ and ${\Delta H}_{i}$ are the chemical, elastic, and internal interface enthalpies changes, respectively.

The chemical enthalpy change is the main contribution to the overall energetics in Equation (14). It is constant throughout the chemically homogeneous specimen and insensitive to the transformation temperature for a given composition. It can be measured through a single interface transformation where no elastic energy is stored as transformational shape change takes place against atmospheric air pressure and accounting for the interface enthalpy.

The elastic enthalpy change is about an order of magnitude smaller than the chemical enthalpy change. In contrast to the chemical enthalpy change, the elastic enthalpy change introduces a temperature dependance to the net enthalpy change because the amount of stored elastic strain energy increases as the transformation proceeds on cooling. In a partially transformed specimen, the elastic strain energy will be inhomogeneous. Elastic enthalpy change can be calculated as follows:

(15) $$\Delta H_{el}=\Delta G_{el}+T\Delta S_{el}$$

where $\Delta G_{el}$, $\Delta S_{el}$, and T are the free elastic energy, change in entropy, and temperature, respectively.

The martensite formed in NiTi alloys is internally twinned. The twin boundaries are of low energy and, therefore, are highly mobile [16]. The friction associated with the movement of the twin-related martensite interfaces causes a change in the internal interface enthalpy. It does not create a significant contribution to the net enthalpy change. However, together with elastic enthalpy, the internal interface enthalpy increases as the transformation proceeds. The magnitude of the internal interface enthalpy change is about two orders of magnitude smaller than the chemical enthalpy change. It can be expressed approximately as [17]

(16) $$\Delta H_{i}=\frac{\gamma V_{m}}{s_{t}}$$

where $\gamma ,V_{m}$, and $s_{t}$ are the twin-interfacial energy, molar volume, and twin spacing, respectively. The elastic enthalpy and internal interface enthalpy contributions are opposite in sign to the chemical enthalpy change. Therefore, they cause a reduction in the total (net) enthalpy change according to Equation (14).

**Figure 6:**
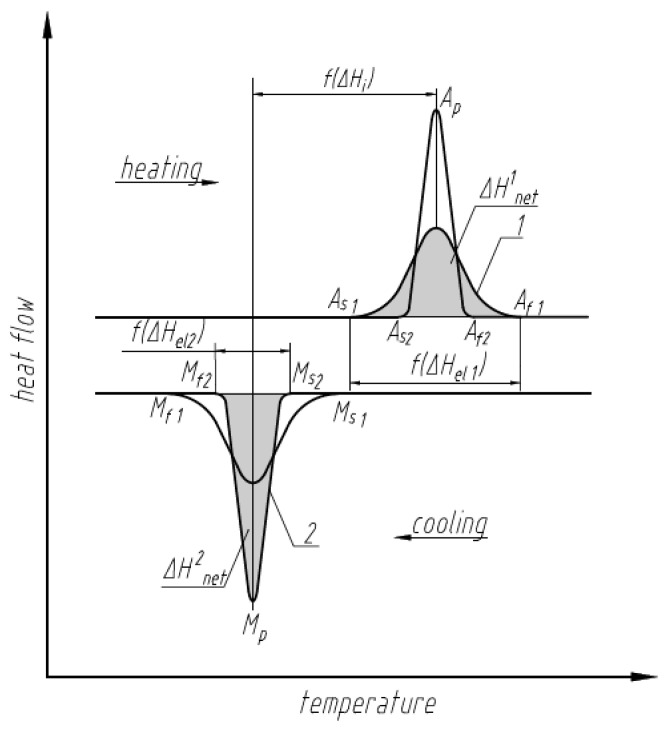
Representative differential scanning calorimetry responses of SMAs with different elastic strain energies.

Hysteresis is a manifestation of the energy losses between the forward and reverse martensitic transformation. Macroscopically, it can be observed by Differential Scanning Calorimetry (DSC) obtained under no applied stress in accordance with ASTM F2082/F2082M-16 [18]. When heating and cooling of an SMA element occurs between the temperature at which the forward transformation to martensite and the reverse transformation to austenite finish, the temperature response of SMAs in DSC curves occurs as a result of the phase transformation. Figure 6 represents a schematic of the DSC responses of two NiTi-based SMA samples. Since the transformation upon heating is endothermic, the amount of associated latent heat should be taken into account for estimation of the required heat for engine activation.

Transformations with different amounts of stored elastic strain energy but with equal amounts of internal interface enthalpy change are shown in Figure 6. The amount of stored elastic strain energy affects the range of the austenite and martensite transformation temperature ranges, i.e., ${(A}_{f}-A_{s})$ and $\left(M_{f}-M_{s}\right)$, and in general is thus associated with the total enthalpy change. In the case where $\Delta {H_{el}}_{1}$ > $\Delta {H_{el}}_{2}$ the range for transformation 1 is wider than the range for transformation 2. Different interfacial friction contributions (i.e., different internal interface enthalpies) in the transformation would affect the relative position of the martensite peak $M_{P}$ and austenite peak $A_{P}$ on DSC curves (Figure 6). In a frictionless transformation, the positions of the peaks would lie on one vertical line, while increased friction will increase the separation and, thus, increase the hysteresis associated with the transformation. The total amount of latent heat associated with the transformation 1 ($\Delta H_{net}^{1}$) will be less then associated with the transformation 2 ($\Delta H_{net}^{2}$) since, in accordance with Equation (14), $\Delta {H_{el}}_{1}$ > $\Delta {H_{el}}_{2}$ [17].

**Figure 7:**
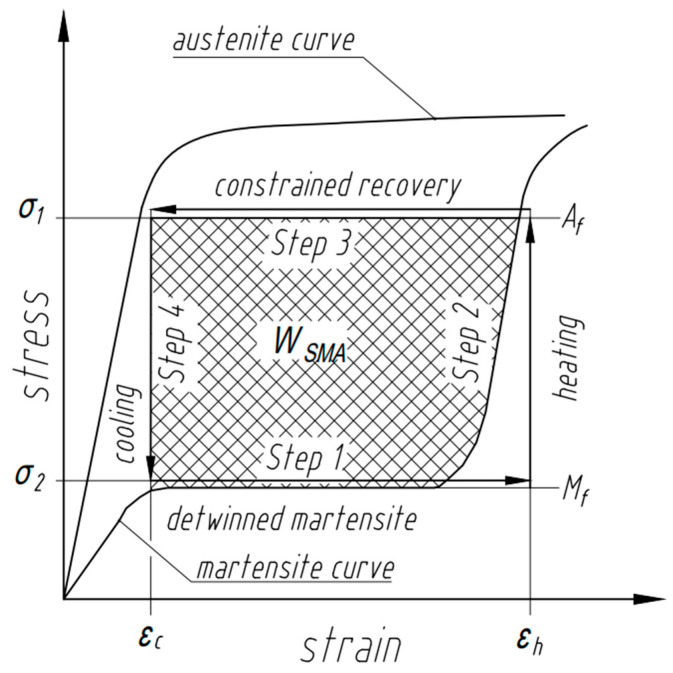
Schematic of SMA work output showing Steps 1 through 4 of the Stirling Cycle from Figure 1.

When sensible heat is added to the system, the temperature of the SMA element changes from $M_{f}^{\sigma_{2}}$ to $A_{f}^{\sigma_{1}}$ being associated with different phases and stresses. The heat associated with the stress-free enthalpy change represents the absorption of latent heat by the alloy and initiates the transformation from pre-deformed martensite to recovered austenite. SMAs produce work only through reverse transformation from martensite to austenite and do not produce substantial work upon cooling [1,12]. The unit work output that an SMA is able to produce in one cycle can be estimated from the area between austenite and martensite curves in the stress-strain graph as illustrated by the cross hatched area shown in Figure 7 and can be expressed as

(17) $$W_{SMA}=\oint \sigma d\varepsilon =\int_{\varepsilon_{c}}^{\varepsilon_{h}}\sigma_{1}d\varepsilon -\int_{\varepsilon_{c}}^{\varepsilon_{h}}\sigma_{2}d\varepsilon \approx \Delta \sigma \cdot \Delta \varepsilon$$

The maximum material work output under $\sigma_{2}$ is at the stress above the twinning stress-plateau of martensite, and the work output under $\sigma_{1}$ is at the stress below plastic yielding in austenite, with the assumption that the martensite forms in temperature space rather than in stress space [19,20].

For the engine considered in this work, from Figure 7, the maximum SMA work output can be achieved by operating between $\varepsilon_{c}$ and $\varepsilon_{h}$, where $\varepsilon_{c}$ corresponds to the onset of detwinning, and $\varepsilon_{h}$ corresponds to the completely detwinned martensitic wire, respectively.

When a bias component is present, part of the work done by the SMA is used to overcome the biasing force $W_{bias}$ [12,16].

(18) $$W_{out}=W_{SMA}-W_{bias}$$

In the specific example of the SMA Stirling engine, biasing force was introduced by the wire bending around the pulleys. Therefore, the magnitude of the useful work is work done by the material to change the shape of the wire under applied stress and strain minus the work done against biasing work from both pulleys and can be estimated in the same way as the output torque in Equation (9).

As seen from Equation (13), Equation (17), and Figure 6 through Figure 7, all three components $Q_{s}$, $\Delta H_{net}$, and $W_{SMA}$ depend on the alloy hysteresis. Therefore, hysteresis is an important factor which determines the amount of required input heat in Equation (12).

In the following, we present a summary of enthalpy contributions to the net enthalpy change in Equation (14) with the objective of re-visiting these contributions subsequently in this work. To analyze energy losses associated with the hysteresis, it is more convenient to express the required heat with elastic and inelastic components. Elastic strain energy arises around the martensite plates formed during the transformation and from the thermal expansion of the material. This contribution is fully recoverable. Inelastic energy is wasted heat which does not produce any useful work. In addition, inelastic energy may also include contributions from a residual martensite phase that nominally would have been expected to transform [21].

Since the martensitic transformation is associated with a shape change, when a martensite plate forms from the parent phase, a large strain arises around the martensite particle. In the case of a thermoelastic transformation, when the transformational shape change is accommodated primarily elastically, the build-up of elastic strain energy opposes further growth of a martensite plate, which was initially driven by the chemical free-energy change [22]. However, the shape change may not be fully accommodated elastically. If, during the transformation, appreciable plastic accommodation by slip takes place, the relaxation of the elastic strain field around the particle will destroy the ideally thermodynamic restoring force responsible for the small transformational hysteresis. To the degree that plastic accommodation takes place, the corresponding decrease in stored elastic energy will require higher energy to reverse the transformation in the SMA with reduced stored elastic energy. Regardless of the amount of stored elastic energy, hysteresis always exists because the main contribution to the hysteresis comes from frictional resistance to the interfacial motion between phases and the compatibility at the austenite/martensite interface. Plastic accommodation of transformation strain will cause a higher dislocation density in the matrix which in a non-cycled sample increases the interfacial friction stress and therefore contributes an additional irreversible component to the overall energetics. Therefore, both considerable frictional resistance during the forward and reverse transformations and amount of stored elastic energy will introduce a deviation from thermoelastic equilibrium.

### 3.3. Material Nano- and Microstructure

In the following, we present the role of nano- and microstructure in affecting SMA heat engine performance in terms of the aforementioned mechanical and thermodynamics aspects, with the objective of re-visiting these contributions subsequently in this work.

The activation energy for homogeneous nucleation of a single variant of martensite is very high to be overcome by a moderate amount of thermal energy [23]. Hence, the internal stress state arising from the nano- and microstructure (both starting and evolving, e.g., [24,25]) plays an even more important role in SMAs. While the grain size is relevant in SMAs for the same reason it is relevant in metallic materials (e.g., grain boundary strengthening to increase the onset of slip or benefits for fatigue/fracture with smaller grain size), it additionally controls the nano- and microstructure through interface area and interface boundary movement during variant reorientation or coalescence (e.g., see evidence of nano twinning in Refs. [26,27] or the observation of compound twins in sub 100 nm grain sizes in Refs. [28,29]). For example, in a transformation that is both single crystal and single interface, there is no storage of elastic strain energy. Additionally, if there is no frictional loss, the DSC curve is expected to have the martensite start and finish temperatures (and, conversely, the austenite start and finish temperatures) collapse on each other. However, in a polycrystalline sample, there is additional elastic strain energy that is stored during the forward transformation and subsequently released during the reverse transformation that affects the overall enthalpy due to the introduction of elastic constraints. Moreover, internal friction separates martensite and austenite peaks. The above effects increase further with finer grain sizes, resulting in a downward trend in transformation temperatures and increasing hysteresis with decreasing grain size [17].

The lattice correspondence between the austenite and martensite lattices is a unique relationship. In NiTi the lattice correspondence between B2 austenite (subscript “A”) and B19’ martensite (subscript “M”) phases has been established to be of the type [30]

(19) $$(001{)}_{M}//{\left(011\right)}_{A},\;{\left[\bar{1}10\right]}_{M}//{\left[\bar{11}1\right]}_{A}$$

From symmetry, Equation (19) can be accomplished by a total of twelve correspondent martensite variants. All these correspondence-variants yield an identical B19’ structure, but they are different in crystallographic orientation. In a self-accommodated structure, any of twelve correspondence-variants can be formed by cooling from the austenite phase. However, during loading, variant clusters initially formed by cooling reorient, grow, or coalesce to form so-called preferred variants which are able to accommodate the applied stress more effectively (a process commonly referred to as detwinning). The size and distribution of these variants starting from the nanoscale can affect both the mechanics and the thermodynamics of the heat engine (e.g., [26,27,28,29,31]). For example, a strain can be determined (e.g., [32]) for each of these variants or variant combinations, and the case where more variants are selected with smaller strains rather than fewer variants are selected with larger strains yields a steeper stress–strain response with implications for the elastic strain energy and, subsequently, the enthalpy of the transformation [33]. The differences in the stress–strain response affect the stresses and strains (Equation (9)) and, thereby, the overall engine performance.

One way to assess the role of dislocations and defects is by examining the equivalent stress–strain response in a thermoelastic transformation that is stress-induced, in the absence of readily available data on cold worked samples during a thermal-induced transformation. Given the reversible nature of the thermoelastic transformation and the equivalence between stress and temperature, the phenomenology is directly comparable. For this, the work of Ref. [34] is considered. The increase in the level of plastic deformation (from 0 to 11%) decreases hysteresis and increases linearity of the stress–strain curve. Moreover, the increased defect density and corresponding stress field create the conditions for favorable selection of martensite variants or result in their coalescence that reduces the overall energy dissipation in the transformation. Classical thermodynamics associates the decrease in hysteresis and increase in linearity in the stress–strain curve with increased elastic enthalpy change, while decreasing frictional resistance of interfacial motion (or energy dissipation)—say, in this case with mechanical cycling with decreased internal interface enthalpy change [34].

The effects of the precipitates on the strength of the B2-matrix and the behavior of the subsequent transformations strongly depend on precipitate microstructure, their size and shape, interparticle spacing, and stress fields and compositional variation around the precipitates. For example, relatively fine precipitates (sub 160 nm), which are not an obstacle to twin boundaries and easily by passed by martensite variants, exhibited higher transformation strain and lower transformation thermal hysteresis compared to samples with relatively larger precipitates that approached several hundred nanometers in size which acted as obstacles to martensite growth, limiting martensite variant and twin size [35,36,37]. The presence of precipitates results in the transformation being highly inhomogeneous. The precipitates are also favorable spots for new martensite plates to nucleate and are obstacles for dislocations to move. In NiTi, there is a need to tailor near-room temperature transformation temperatures for the end application that can result in Ni_4_Ti_3_ precipitates. These can be different levels of coherency and, therefore, influence the thermodynamics and transformation behavior in different ways. Coherent Ni_4_Ti_3_ restricts complete detwinning of the martensite, resulting in a two-phase structure with partially detwinned martensite and precipitates [38]. Moreover, precipitates increase the stored elastic strain energy and lower transformation temperatures compared to overaged or solutionized NiTi. Strong stress field associated with both coherent and semi-coherent precipitates due to mismatch in lattice parameters between precipitates and matrix prevent small local areas of the specimen from transforming back to austenite. In high-temperature shape memory alloys (HTSMA) (e.g., in cases where such alloys are used in heat recovery applications) NiTiPt and NiTiHf, nanosized precipitates of so-called P-phase and H-phase have been identified [39,40]. Both P- and H-phases are fully coherent precipitates embedded in the B2-matrix. P-phase is introduced in the (Ni,Pt)Ti system by aging the material at a relatively high temperature (~600 °C) for a relatively long time (~100 h). P-phase precipitates have a monoclinic structure (Ti_44_Ni_36_Pt_16_, a_p_ = 0.745 nm, b_p_ = 1.292 nm, c_p_ = 1.422 nm and 𝛽 = 100.45 [39]) and have near cuboidal shape with edge length about 200–400 nm and faces nearly parallel to planes {100}. The precipitation causes changes in concentration in the B2-matrix due to depletion of Ni while enriching Pt. Both the chemical non-uniformity and stress field associated with the P-phase precipitates are in favor of the martensitic transformation and affect the martensite start temperature. Moreover, the internal friction also strongly depends on precipitate size, shape, and spacing. The changes in (Ni,Pt)Ti system thermodynamic state associated with precipitation of P-phase particles not only change the net enthalpy change but also increase the martensite start temperature. Based on experimental measurements, it results in $M_{s}$ increasing by 100 °C [39].

H-phase precipitates in the Ni-(Ti,Hf) system (stoichiometry about Ni_50_(Ti,Hf)_50_) are nano-size coherent particles which appear in the B2 matrix. The equilibrium shape of the H-phase precipitates is a diamond-like shape with the longest dimension of the particle around 10 nm. The state can be achieved by aging Ni-(Ti,Hf) alloy at a relatively low temperature (e.g., 550 °C) for a relatively short time (e.g., 3 h). Aging at a relatively high temperature (e.g., 600 °C) for a relatively long time (e.g., 815 h) transforms the equilibrium particle shape to a thin plate shape with increased length of the longest dimension to around 500 nm. H-phase precipitates are of B19 orthorhombic structure with lattice parameters a_h_ = 1.264 nm, b_h_ = 0.882 nm, c_h_ = 2.608 nm [40]. Due to changes in the B2-B19 symmetry, only six correspondence variants of the H-phase are possible. As with the case of the P-phase, the H-phase precipitation process activates phenomena which affect the strength of the B2 matrix and the behavior of the subsequent martensitic transformation. First, it creates a spatially inhomogeneous stress field around the H-phase particles. Second, because in the Ni-(Ti,Hf) system, the martensite start transformation temperature strongly correlates with the concentration of Ni and Hf in the B2-matrix, growth of H-phase particles creates a concentration gradient which affects martensitic transformation by around 40–60 °C. Third, as expected, the shape and size of precipitates affect interfacial friction during the transformation. The thermal hysteresis is found to increase for higher aging treatment (650 °C, for 3 h) but decreases for lower aging treatment (550 °C, for 3 h). Furthermore, functional fatigue resistance improves significantly. All these effects have been attributed to different stress and concentration fields around H-phase precipitates in the B2 matrix [40].

## 4. Implementation

Previous work has largely been limited to examining the efficiency of SMA heat engines in the context of the Carnot efficiency or the power output. This would be appropriate when considering other competing technologies when there are no major design constraints, and the application calls for maximizing heat transfer. However, in cases where the energy would be wasted if not recovered (e.g., heat in engines or batteries) or energy that is available due to changes in the ambient temperature (e.g., day/night cycles or even the temperature inside a car on a hot day), the advantages of an SMA heat engine become obvious. As previously stated, these include eliminating the need for external sensors and actuators since the SMA acts as both sensor and actuator with favorable power to weight ratios, stresses, strains, and displacements, thus making the SMA heat engine more compact and reliable. In the following, we go further and propose optimizing the SMA heat engine overall for either torque output or specifically for speed. While an application such as storing energy in a flywheel or a dynamo may rely merely on the overall power output or torque under certain conditions, one that stores the energy in a supercapacitor that depends on charging cycles or an application where functional fatigue limitations of the SMA need to be considered will require specifical control of speed. Fatigue considerations were beyond the scope of this paper, but a million functional cycles are not uncommon in applications [5].

The total output power $P_{out}$ of an engine can be determined from the product of the output torque $T_{out}$ and the output angular speed $\omega_{out}$:

(20) $$P_{out}=T_{out}\cdot \omega_{out}$$

From Equation (9), an increase in $T_{out}$ can be achieved by increasing the wire diameter (or, in the general case, an increase in the section modulus), the radius of the cold pulley $R_{c}$, bending stresses (moments) acting over the cross section of the SMA element after exiting the heat source and following contact of the wire with the cold pulley ($\sigma_{h,exit}{,\;\sigma }_{c,c}$, respectively, from Figure 4), or by decreasing the bending stresses (moments) acting over the cross section of the SMA element prior to entering the heat source and first contact of the wire with the cold pulley ($\sigma_{h,entr},\sigma_{c,h}$, respectively from Figure 4) and the radius of the hot pulley $R_{h}$. It is possible to consider equivalent strains rather than stresses at the same locations, and this requires maximizing $\varepsilon_{h_{A}}$ and $\varepsilon_{c}$ while minimizing $\varepsilon_{h}$ and $\varepsilon_{c_{A}}$ from Figure 5 in order to increase the torque output.

Figure 4 and Figure 5 illustrate the stress and strain distribution, respectively, in the SMA wire at different temperatures with position. The relevant temperatures $T_{exit}$, $T_{entr},$ $T_{c,h}$, and $T_{c,c}$ increase with an increase in the heat source temperature. However, due to the non-linear stress–temperature and strain–temperature curves, when the wire transforms to the austenite state, stresses and strains are constant in the wire at the hot pulley end even with further increase in temperature of the wire. Further increase in the temperature of the wire due to an increase in temperature of the heat source decreases the stress difference that drives the engine. Thus, a continuous increase in the temperature of the heat source will decrease the output torque.

Following Figure 7, an increase in hysteresis in the alloy implies an increased capability to deliver additional torque. In terms of the latent heat of the transformation, increased net enthalpy change and increased amount of stored elastic strain energy, which affects the austenite and martensite transformation temperature ranges, will also be beneficial. However, the net enthalpy change and elastic strain energy are competing contributions in Equation (14). Therefore, further assessment for the particular case with quantitative analyses for the relative contributions following this framework are required.

Applying Equation (20) to the unsynchronized engine considered in the current work, the angular speed of the cold pulley is the output speed. Multiplying maximum angular speed from Equation (11) by the transmission ratio $\frac{R_{h}}{R_{c}}$, the maximum angular speed of the cold pulley can be obtained

(21) $$\omega_{c_{max}}=-\frac{4\cdot h\cdot \varphi }{\rho \cdot c_{p}\cdot d\cdot ln\left(\frac{T_{so}-A_{f}}{T_{so}-M_{f}}\right)}\cdot \frac{R_{h}}{R_{c}}$$

For simplicity, the difference in heat transfer coefficients between the SMA element and heat source or heat sink is neglected in the subject framework but will need to be considered for more rigorous heat transfer analysis.

From Equation (21), an increase in $\omega_{c_{max}}$ can be achieved by decreasing the wire diameter (or, in the general case, decreasing the section modulus) and radius of the engine cold pulley $R_{c}$, decreasing the thermal hysteresis, or by increasing the radius of the hot pulley $R_{h}$ and increasing the temperature of the source.

To quantitatively estimate the performance of SMA elements in SMA heat engines regardless of the working temperature range, component size, and mass but considering the need for elevated torque or speed, a relative measure of the material performance with the proposed Figure of Merit (FOM) can be employed. The FOM is a convenient tool which can predict the relative output of the SMA element during the design and development of the heat engine depending on the application requirement.

From a discussion of the parameters affecting SMA microstructure, and considering the previously proposed FOM [41] for phase change materials in general, the material efficiency for SMA heat engine applications requiring either high torque or high speed can be estimated from the FOM presented in Equation (22):

(22) $$FOM=\frac{\Delta H_{net}\cdot c_{p}\cdot \left|A_{f}-M_{f}\right|}{\rho \cdot k}$$

where ρ, $\Delta H_{net}$, k, $c_{p},$ and $A_{f}-M_{f}$ are the density, the latent heat of transformation, the thermal conductivity of the SMA, specific heat capacity, and thermal hysteresis, respectively.

The FOM represents a measure of the volumetric capacity of SMAs to recover energy stored in a system by activating the transformation in an SMA element of a heat engine with a fixed amount of heat. For increased output torque of an SMA Stirling engine, the FOM should be as high as possible, while for applications requiring maximum output speed, it should be as low as possible. A high torque is favored by high enthalpy, high specific heat, a wider thermal hysteresis, and additional time for the SMA to reach steady state while high speed is inversely favored by low enthalpy, low specific heat, a narrower thermal hysteresis, and a faster approach to steady state conditions.

Based on the aforementioned presentation, we now examine specific aspects of implementing the framework. The transformation temperatures in SMAs are highly sensitive to the chemical composition of the alloy. Therefore, changing the stoichiometric composition of the alloy or alloying with ternary or quaternary elements can modify the transformation temperatures and help select the alloy for use at appropriate temperatures depending on the application (e.g., [3]).

In binary NiTi alloy, the highest $A_{f}$ is in a 50 at % Ti-50 at % Ni alloy and is about 120 °C. The general trend is that the shape recovery temperature decreases with increasing Ni content and does not change with decreasing Ni. Pt and Hf are elements that introduce P- and H-phases, respectively, and as previously discussed, not only raise the transformation temperatures but also change the hysteresis width. Ph and Zr also elevate transformation temperatures. In Ti-Ni-Cu alloys, with an increase in the Cu content, the transformation hysteresis decreases. In contrast, addition of Nb broadens the transformation hysteresis [1]. The substitution of Co for Ni lowers the transformation temperatures. Addition of elements such as Fe (substitution for Ni in NiTi) not only lowers the martensite finish temperature down to −153 °C but also introduces an intermediate trigonal R-phase which separates the complete martensitic transformation into two: B2→R-phase and R-phase → B19’.

The R-phase transformation is associated with a narrow temperature hysteresis of 1.5 °C and with a maximum recoverable strain of only about 1%, while the hysteresis associated with the B2 → B19’ transformation exceeds 10 °C and possesses about 8% recoverable strain while exhibiting shape memory behavior [16]. The fatigue life for the R-phase is high compared to the full B19’ phase transformation [42]. Since the transformation through an intermediate R-phase transition and a direct austenite martensitic transformation are competing processes, an R-phase transformation can be achieved by suppressing the B2 → B19’ transformation. The R-phase transition can be attributed to the presence of previously discussed Ni_4_Ti_3_ precipitates in the alloy B2 matrix. The presence of the precipitates produces a strong resistance to large lattice variant transformation associated with the formation of B19’ [16]. The transformation through the intermediate trigonal R-phase produces a significantly smaller lattice variant transformation and is much less affected by Ni_4_Ti_3_ precipitates. Ref. [42] showed that R-phase transformation temperatures can be decreased to cryogenic temperatures by increasing Ni/Ti ratios in NiTiFe. Even though the addition of Fe as a ternary element introduces the R-phase, no phase transformation is observed in the Ti-rich alloys with more than 4 at % Fe. Ni-rich alloys with 4 at % Fe exhibit the R-phase transformation only after thermo-mechanical treatments and multiple thermo-mechanical treatments (annealing+ cold working or solutionizing + quenching + cold working + annealing). Thermo-mechanical treatments affect the size and distribution of Ni_4_Ti_3_ precipitates. In general, the size of the meta-stable Ni_4_Ti_3_ precipitates decreases with increasing Fe content. The ability to operate at cryogenic temperatures with low hysteresis and increased fatigue life makes the R-phase an attractive choice for SMA engines employed for rejecting parasitic heat in space applications. However, taking into consideration the amount of latent heat involved in the transformation through the R-phase vs. B19’ for maximizing heat rejection within a fixed period of engine operation is not straightforward. From quantitative results obtained for NiTi in Ref. [41], the martensite to austenite phase transformation absorbs three times more energy (latent heat) and provides three times higher temperature suppression than the R-phase to austenite transition, while also having a higher FOM and, thus, lower angular speed. Considering the sensitivity of the SMA to composition and thermo-mechanical treatment, the choice between transformation through R-phase or directly to B19’ for maximizing heat rejection can be analyzed further for the particular material and design parameters using the framework presented.

The demonstration engine evaluated in this work operated between hot water, and the ambient temperature and experimentally cooling were observed to be a slower process compared with heating. Therefore, analogous to Equation (10), the cooling time and, subsequently, the rotation speed are governed by

(23) $$\tau_{sink}=-\frac{\rho \cdot c_{p}\cdot d}{4h}\ln\left(\frac{T_{sink}-M_{f}}{T_{sink}-A_{f}}\right)$$

where the temperature of the wire after exiting the heat source is $A_{f}$ and the temperature of the heat sink is $T_{sink}$. Therefore, following Figure 2, the distance between pulleys D sets the engine size, and the wire length should be assigned based on heat transfer conditions in such way that after leaving the heat source zone, the heated segment of the SMA wire (at temperature ≥ $A_{f}$) should have enough time to be cooled down to temperature $M_{f}$ in the heat sink before starting the next heating cycle.

To activate cycling of the phase transformation between high and low temperatures following Figure 1, the external moment should introduce an unbalanced state in the static equilibrium of the engine. This can be done with two different approaches: first, by design with mechanical actuation, e.g., offset axis design [6] which introduces a difference between the center of gravity and the center of rotation and thereby creates a starting moment; second, a higher temperature of the heat source results in a heterogeneous transformation in the SMA element due to non-steady state heat transfer conditions that produce an unbalanced stress state to start the engine. This heterogenous transformation has previously been observed experimentally [43,44]. Of note is the ultrafine (50 nm) grained structure in the sample in Ref. [44] which was verified in Ref. [45]. This was qualitatively validated with the demonstration engine—lower temperatures of the hot water required external mechanical force to start the engine no matter how long it was held at that temperature in the hot water. However, increasing the temperature of the hot water resulted in the engine starting itself without the need for an external force. Hence, the transient response prior to steady state conditions helps with the engine starting on its own due to inhomogeneous transformation. As previously mentioned, precipitates also have a similar effect as they make the transformation more inhomogeneous.

As was pointed out with Equation (19), the lattice correspondence between martensite variants and austenite is responsible for the directional dependence of transformation or detwinning strains in martensite and, thus, for the development of favorable nano- and microstructure in NiTi. The evolving and resulting variant nano- and microstructure satisfies stress and strain compatibility at all times. Thus, any way to tailor the internal stress state and the subsequent variant selection affects material behavior for an SMA heat engine. Starting texture or preferred orientation, training, and thermomechanical cycling are ways that bias the overall variant selection and coalescence process. The amount of strain recovery can be optimized by texturizing SMAs for a particular application. However, it also should be considered that a textured sample has lower stored elastic energy in comparison with a sample with randomly oriented grains. This directional dependence may be employed for engineering an SMA alloy for customizing SMA heat engine output through the FOM in Equation (23). Bending is one of the most common loading modes of SMA in engines, including the SMA heat engine considered here. Bending can be considered as tension and compression acting together in one as described previously [46,47].

The sample trained to remember both shapes of austenite and martensite, or the so-called two-way shape memory effect (TWSME), removes the need for biasing mechanisms in the system in accordance with Equation (18). TWSME training causes an increase in the stored elastic energy. However, the small force for the shape change in the forward transformation compared to that in the reverse transformation should be carefully considered in the SMA heat engine design. The TWSME influences the thermodynamics through texture and re-distributing internal stresses more than resulting in a strong biasing force.

In addition, plastic deformation also results in increased residual or nonrecoverable strain. However, it can be significantly reduced by cycling. Cycling provides stabilization of additional martensite (through evolving texture) by inducing plastic deformation. Stress-induced martensite can accommodate the mismatch with martensite that is stabilized from stresses associated with the residual plastic strain. The mismatch is fully accommodated elastically following cycling. However, prior to this elastic accommodation, the initial cycles involve inelastic accommodation, which is associated with phase fraction and texture evolution through twinning in martensite [34,48,49].

Bulk nucleation introduces additional constraints to the system and increases the elastic strain enthalpy change. Following Figure 6, it increases the difference in temperatures between the start and finish of the austenite and martensite phase transformation and, in accordance with Equation (14), decreases the net enthalpy change. Oxide and nitride coatings of the SMA elements can be used to inhibit surface nucleation and achieve bulk nucleation based on similar observations in Ni-plated NiTi [17]. Thick wire will also be a way to promote bulk nucleation. In contrast, for surface nucleation, the temperatures of start and finish for each phase would be located closer to each other, and the net enthalpy change will be higher. A cable consisting of bundle wires with high surface area exposed for surface nucleation will be a preferable configuration from the design side to achieve surface nucleation. However, as previously outlined, the net enthalpy change and the elastic enthalpy change are competing contributions. The sum contribution can be analyzed further for the particular material and design parameters using the framework presented.

## 5. Conclusions

While SMA Stirling heat engines have been around for some time, to the best of our knowledge, there has not been a combined examination of thermal, mechanical, and materials aspects with a framework for their design and implementation until this work. Furthermore, previous analyses have merely considered an overall Carnot efficiency without taking into consideration that SMA heat engines are advantageous and can run with heat scavenged from sources that are nearly limitless or are otherwise even wasted. Taking into consideration end applications for thermal management and energy recovery, the framework proposed distinguishes between a high speed and a high torque mode of performance in contrast with a more traditional power or a total amount of heat transferred criterion. We present the following summary:

1. Mechanical aspects in the framework were built on force balances in the SMA element and on the resulting stress distribution. The role of element geometry was assessed in affecting engine output torque and speed from the analysis of the SMA Stirling engine.
2. Thermal aspects were addressed by considering the heat transfer rate between the SMA element and both the heat source and the heat sink. The effect of temperature of the heat source relative to the phase transformation temperatures of the SMA was also evaluated.
3. The total enthalpy of the transformation was considered from chemical, elastic, and frictional contributions. Each of these terms was connected with the material nano- and microstructure through alloy selection and thermomechanical processing, making recommendations vis-á-vis the size and distribution of precipitates, variant interfaces through texture, cycling and training, defects, nucleation sites (bulk vs. surface), and multi-step transformations (e.g., a trigonal R-phase transformation). Specific cases of tailoring P-phase and H-phase nano-precipitates in NiTiPt and NiTiHf high temperature shape memory alloys (for heat recovery applications) affecting nano- and compound twinning and, subsequently, the enthalpy of the transformation by recourse to ultra fine and nano crystalline grain structures and additionally introducing a inhomogeneous transformation in ultra fine grained materials for adequate starting torque in order for the engine to run without external force, are presented.
4. The above aspects were extended to consider a figure of merit for the performance of an SMA heat engine in two modes of operation. The first is where torque is the defining criterion, and the second is where speed is. The importance of separately using both an enthalpy term and a hysteresis term in the proposed FOM is emphasized through competition between the net enthalpy change and the elastic enthalpy change.
5. For an SMA heat engine functioning where maximum torque is the criterion, a high FOM is a desirable choice. In contrast to torque, for an SMA heat engine functioning where maximum speed is the criterion, a recommendation on minimizing the FOM is made.

The purpose of this work was to present a theoretical framework of an SMA Stirling heat engine. The working demonstration was used to qualitatively identify and validate important issues. A more sophisticated and instrumented implementation of the demonstration model is being undertaken to facilitate quantitative analyses of the framework in order to raise the technology readiness of level of SMA Stirling heat engines for thermal management and energy recovery applications.

## Data Availability

Not applicable.
